# The Complete Mitochondrial Genome of the Caecal Fluke of Poultry, *Postharmostomum commutatum*, as the First Representative from the Superfamily Brachylaimoidea

**DOI:** 10.3389/fgene.2019.01037

**Published:** 2019-10-25

**Authors:** Yi-Tian Fu, Yuan-Chun Jin, Guo-Hua Liu

**Affiliations:** ^1^College of Veterinary Medicine, Hunan Agricultural University, Changsha, China; ^2^Hunan Co-Innovation Center of Animal Production Safety, Changsha, China

**Keywords:** *Postharmostomum commutatum*, mitochondrial genome, mitochondrial DNA, phylogenetic analyses, paraphyly

## Abstract

*Postharmostomum commutatum* (Platyhelminthes: Brachylaimoidea), a parasite of the caeca of poultry, has been frequently reported from many countries and regions, including China. However, the molecular epidemiology, population genetics and phylogenetics of this parasite are poorly understood. In the present study, we determined and characterized the complete mitochondrial (mt) genome of *P. commutatum*, as the first representative from the superfamily Brachylaimoidea. The mt genome of *P. commutatum* is a circular DNA molecule of 13,799 bp in size and encodes the complete set of 36 genes (12 protein-coding genes, 22 transfer RNA genes, two ribosomal RNA genes) as well as a typical control region. The mt genome of *P. commutatum* presents a clear bias in nucleotide composition with a negative AT-skew on average (−0.306) and a positive GC-skew on average (0.466). Phylogenetic analyses showed that *P. commutatum* (superfamily Brachylaimoidea) and other ten members of the order Diplostomida were recovered as sister groups of the order Plagiorchiida, indicating that the order Diplostomida is paraphyletic. This is the first mt genome of any member of the superfamily Brachylaimoidea and should represent a rich source of genetic markers for molecular epidemiological, population genetic and phylogenetic studies of parasitic flukes of socio-economic importance in poultry.

## Introduction

*Postharmostomum commutatum* (= *P. gallinum*) (Platyhelminthes: Brachylaimoidea) is one of the most common flukes of poultry (Taylor et al., 2016). This parasite inhabits the intestinal caeca of poultry and may be associated with the occurrence of inflammation and hemorrhages in heavily infected animals (Taylor et al., 2016). *P. commutatum* was mentioned for the first time by Wagener (1852), who found this parasite in the caeca of a young chicken from Italy. To date, this parasite has been frequently reported in Africa, the Americas, Asia and Europe (Valadão et al., 2018).

Metazoan mitochondrial (mt) genome is a biological macromolecule containing 36–37 genes (12–13 protein-coding genes, two ribosomal RNA genes and 22 transfer RNA genes) (Boore, 1999; Hu and Gasser, 2006). This gene content has been shown to vary in cestodes and trematodes and Chromadorea nematodes, which lack *atp*8 gene (Boore, 1999). Due to its maternal inheritance, high genome copy numbers, fast evolutionary rate, simple genetic structure and lack of recombination, mt genome sequences have been widely used in molecular epidemiological, population genetic and phylogenetic studies at various taxonomic levels of different parasitic worms (Zhang et al., 2017; Wu et al., 2018; Jin et al., 2019).

The digeneans (subclass Digenea) are distributed worldwide and comprise ∼18,000 described species (Kostadinova and Pérez-Del-Olmo, 2019). The phylogeny and classification of digeneans have been substantially revised with analyses of the two nuclear ribosomal RNA genes (Olson et al., 2003; Littlewood et al., 2015; Pérez-Ponce de León and Hernández-Mena, 2019). Recently, mt genomic datasets have also been used to understand the phylogenetic relationships of digeneans (Webster and Littlewood, 2012; Brabec et al., 2015; Briscoe et al., 2016; Chen et al., 2016; Locke et al., 2018). A major difference between the most accepted classification based on nuclear rRNA genes and mt genome phylogenies is the order Diplostomida. All previous phylogenetic analyses based on nuclear rRNA genes have supported the monophyly of this order within the subclass Digenea (Olson et al., 2003; Littlewood et al., 2015; Pérez-Ponce de León and Hernández-Mena, 2019). However, mt genome phylogeny rejected the monophyly of this order because *Clinostomum complanatum* (Schistosomatoidea), *Alaria americana*, *Hysteromorpha triloba*, *Tylodelphys immer*, *Cardiocephaloides medioconiger*, *Cotylurus marcogliesei*, *Posthodiplostomum centrarchid*, *Cyathocotyle prussica* (Diplostomoidea) and two *Diplostomum* species were recovered as sister groups of order Plagiorchiida, not the order Diplostomida (Brabec et al., 2015; Chen et al., 2016; Locke et al., 2018). The order Diplostomida currently consists of three superfamilies (Brachylaimoidea, Diplostomoidea and Schistosomatoidea). The superfamily Brachylaimoidea has potential veterinary importance and complex taxonomic history. Currently, the superfamily Brachylaimoidea contains at least 20 valid species that consist of parasites of mammals and birds (Heneberg et al., 2016). Despite their importance, no mt genome had been sequenced and characterized for any members of the superfamily Brachylaimoidea.

The objectives of the present study were to determine and analyze the complete mt genome of *P. commutatum*, as the first representative from the superfamily Brachylaimoidea, and to assess the systematic and phylogenetic position of this fluke within the subclass Digenea using concatenated protein sequences derived from all coding genes.

## Materials and Methods

### Parasites and Total Genomic DNA Isolation

Adult specimens of *P. commutatum* were collected from a naturally infected chicken in Hunan province of China. Adult worm specimens were washed separately in physiological saline, identified preliminarily to species based on morphological features described previously (Pojmańska, 2002), fixed in 70% (v/v) ethanol and stored at −20°C until further use. DNA extraction was performed from individual flukes using a commercially available kit (Wizard® SV Genomic DNA Purification System, Promega) according to the manufacturer’s instructions. The molecular identity of each specimen was further verified by PCR using an established method and then sequenced (Bowles and McManus, 1994). The mt *cox*1 sequences of *P. commutatum* samples showed 99% similarity with that of *P. commutatum* from *Gallus gallus* in Brazil (GenBank accession no. MH919409) (Valadão et al., 2018). Our phylogenetic analyses based on mt *cox*1 sequences of *P. commutatum* and relatives showed that two *P. commutatum* isolates grouped together, suggesting that the *Postharmostomum* isolate from present study represented *P. commutatum* (**Figure S1**).

### Sequencing and Assembling

High molecular weight genomic DNA was extracted from an adult fluke and agarose-gel electrophoresis (1%) was used to verify DNA integrity. After fragmentation (400–500 bp) of this DNA by shearing using G-tubes (Coavris M220), a paired-end genomic library (about 320 bp inserts) was constructed using TruSeq™ DNA Sample Prep Kit (Illumina). All sequencing was carried out on Illumina Hiseq 4000 platform and data recorded in FASTQ format. The clean reads were obtained from raw reads by removing adaptor sequences, highly redundant sequences, reads that contained more than 10% ambiguous positions (N) and low-quality reads. Clean reads were assembled into contigs with Geneious 11.1.5 (Kearse et al., 2012) based on mt *cox*1 conserved sequence motifs. The assembly parameters were minimum overlap identity 99.5%, minimum overlap 150 bp and maximum gap size 5 bp. The assembly generated a large contig ending with overlapping fragments. As this structure allowed a single circular organization of the mt genome, we assumed that the complete mt genome had been assembled. The completeness of the mt genome assembly was further verified by long PCR experiment using five pairs of primers (**Table S1**) which were designed in the conserved regions.

### Annotations

The assembled mt genome was annotated with the MITOS webserver (Bernt et al., 2013). The boundaries of protein-coding genes and rRNA genes were determined by alignment with the homologous genes of *C. complanatum* using the computer program MAFFT 7.122 with the option (L-INS-I) (Katoh and Standley, 2013). Amino acid sequences of 12 protein-coding genes were inferred using MEGA 6.0 (Tamura et al., 2013). Translation start and stop codons were identified based on comparison with those of *C. complanatum* reported previously (Chen et al., 2016). The identification, boundary delimitation and secondary structure folding of 22 tRNA genes were identified using ARWEN (Laslett and Canbäck, 2008) and the program tRNAscan-SE (Lowe and Chan, 2016) under the default search model, with the “other mitochondrial” sequence source and the “invertebrate mitochondrial” genetic code, and manual adjustment. The Ka/Ks ratio was calculated for nucleotide sequences of all 12 mt protein-coding genes of *P. commutatum* and other digeneans using DnaSP v5 (Librado and Rozas, 2009).

### Phylogenetic Analysis

All mt genome sequences of subclass Digenea (**Table 1**), along with an outgroup of the subclass Monogenea (*Gyrodactylus derjavinoides*; GenBank accession number EU293891) (Huyse et al., 2008), were obtained from GenBank and combined for phylogenetic analysis. The deduced amino acid sequences of 12 protein-coding genes were aligned individually using MAFFT 7.122. The well-aligned conserved blocks were identified using Gblocks 0.91b with default parameters using the option for a less stringent selection (Talavera and Castresana, 2007). The individual amino acid or concatenated amino acid alignments and newick trees have been stored in a publicly available data repository (Accession ID: 25084; Study Accession URL: http://purl.org/phylo/treebase/phylows/study/TB2:S25084). Phylogenetic analyses were conducted using Maximum likelihood (ML) and Bayesian inference (BI). ML analysis were computed using PhyML 3.0 (Guindon et al., 2010). For ML analysis, it was partitioned by gene, and bootstrapping frequencies (BS) was performed using the rapid bootstrapping option with 100 iterations, the JTT (genes 1–6; *cyt*b, *cox*3, *nad*2, *nad*4L, *nad*5 and *nad*6), LG (genes 7–8; *cox*2 and *nad*4), MtArt (genes 9–10; *cox*1 and *nad*1) and MtREV (genes 11–12; *atp*6 and *nad*3) models were used as selected by ProtTest 2.4 (Abascal et al., 2005) based on the Akaike information criterion (AIC). BI analysis was performed using MrBayes 3.2.6 (Ronquist and Huelsenbeck, 2003), two independent runs with four incrementally heated Metropolis-coupled Markov chains Monte Carlo were run for two million generations, with tree sampling conducted at every 200 generations. The first 25% of the sampled trees were discarded as burn-in, and the remaining trees were used to calculate Bayesian posterior probabilities (Bpp). The potential scale reduction factor approached 1 and the average split frequency of less than 0.01 were used to represent the convergence of the two simultaneous runs. For BI analysis, the dataset was partitioned by gene, and the amino acid model for each gene was estimated from above models with model-given frequencies and gamma distributed rates. PhyloBayes 3.3b (Lartillot and Philippe, 2004) was run using the site-heterogeneous mixture CAT model, and the analysis was stopped when the conditions considered to indicate a good run were reached (maxdiff <0.1 and minimum effective size >300). The phylogenetic trees were visualized using FigTree v.1.42 (http://tree.bio.ed.ac.uk/software/figtree/). 

**Table 1:** Mitochondrial genome sequences of digeneans sequenced completely prior to the present study and used for phylogenetic analysis.

Order	Family	Species	Size (bp)	GenBank accession number
Diplostomida	Cyathocotylidae	*Cyathocotyle prussica*	13,665	NC_039780
	Clinostomidae Diplostomidae	*Clinostomum complanatum* *Alaria Americana* *Diplostomum pseudospathaceum* *Diplostomum spathaceum* *Hysteromorpha triloba* *Posthodiplostomum centrarchid* *Tylodelphys immer*	13,796 13,836 14,099 14,784 13,855 14,561 14,193	NC_027082 MH536507 KR269764 KR269763 MH536511 MH536512 MH536513
	Schistosomatidae	*Schistosoma spindale*	16,901	DQ157223
		*Schistosoma haematobium*	15,003	DQ157222
		*Schistosoma japonicum*	14,087	JQ781215
		*Schistosoma mansoni*	14,415	NC_002545
		*Schistosoma mekongi*	14,072	AF217449
		*Schistosoma margrebowiei*	15,167	AP017709
		*Trichobilharzia szidat*	14,293	NC_036411
	Strigeidae	*Trichobilharzia regent* *Cardiocephaloides medioconiger* *Cotylurus marcogliesei*	14,838 15,107 13,815	DQ859919 MH536508 MH536509
Plagiorchiida	Brachycladiidae	*Brachycladium goliath*	15,229	NC_029757
	Dicrocoeliidae	*Dicrocoelium dendriticum*	14,884	NC_025280
		*Dicrocoelium chinensis*	14,917	NC_025279
		*Eurytrema pancreaticum*	15,031	KP241855
	Echinochasmidae	*Echinochasmus japonicus*	15,865	NC_030518
	Echinostomatidae	*Artyfechinostomum sufrartyfex*	14,567	NC_037150
		*Echinostoma caproni*	14,150	AP017706
		*Isthmiophora hortense*	14,994	KR062182
		*Echinostoma miyagawai*	14,416	NC_039532
		*Hypoderaeum sp.*	14,180	KM111525
		*Echinostoma paraensei*	20,298	KT008005
	Fasciolidae	*Fasciola gigantica*	14,478	KF543342
		*Fasciola hepatica*	14,462	AF216697
		*Fasciola jacksoni*	14,952	KX787886
		*Fascioloides magna*	14,047	NC_029481
		*Fasciolopsis buski*	14,833	NC_030528
	Gastrodiscidae	*Homalogaster paloniae*	14,490	KT266674
	Gastrothylacidae	*Gastrothylax crumenifer*	14,801	NC_027833
		*Fischoederius cobboldi*	14,256	KX169164
		*Fischoederius elongatus*	14,120	NC_028001
	Heterophyidae	*Haplorchis taichui*	15,130	NC_022433
		*Metagonimus yokogawai*	15,258	NC_023249
	Himasthlidae	*Acanthoparyphium sp.*	14,191	MG792058
	Notocotylidae	*Ogmocotyle sikae*	14,307	NC_027112
	Opisthorchiidae	*Metorchis orientalis*	13,834	NC_028008
		*Opisthorchis felineu*	14,277	EU921260
		*Clonorchis sinensis*	13,875	FJ381664
		*Opisthorchis viverrini*	13,510	JF739555
	Paramphistomidae	*Paramphistomum cervi*	14,023	KT198987
		*Calicophoron microbothrioides*	14,028	NC_027271
		*Explanatum explanatum*	13,968	NC_027958
		*Orthocoelium streptocoelium*	13,800	KM659177
	Troglotrematidae	*Paragonimus heterotremus*	13,927	NC_039430
		*Paragonimus ohirai*	14,818	NC_032032
		*Paragonimus westermani*	14,103	NC_027673

## Results and Discussion

### Genome Organization and Composition

**Figure 1:**
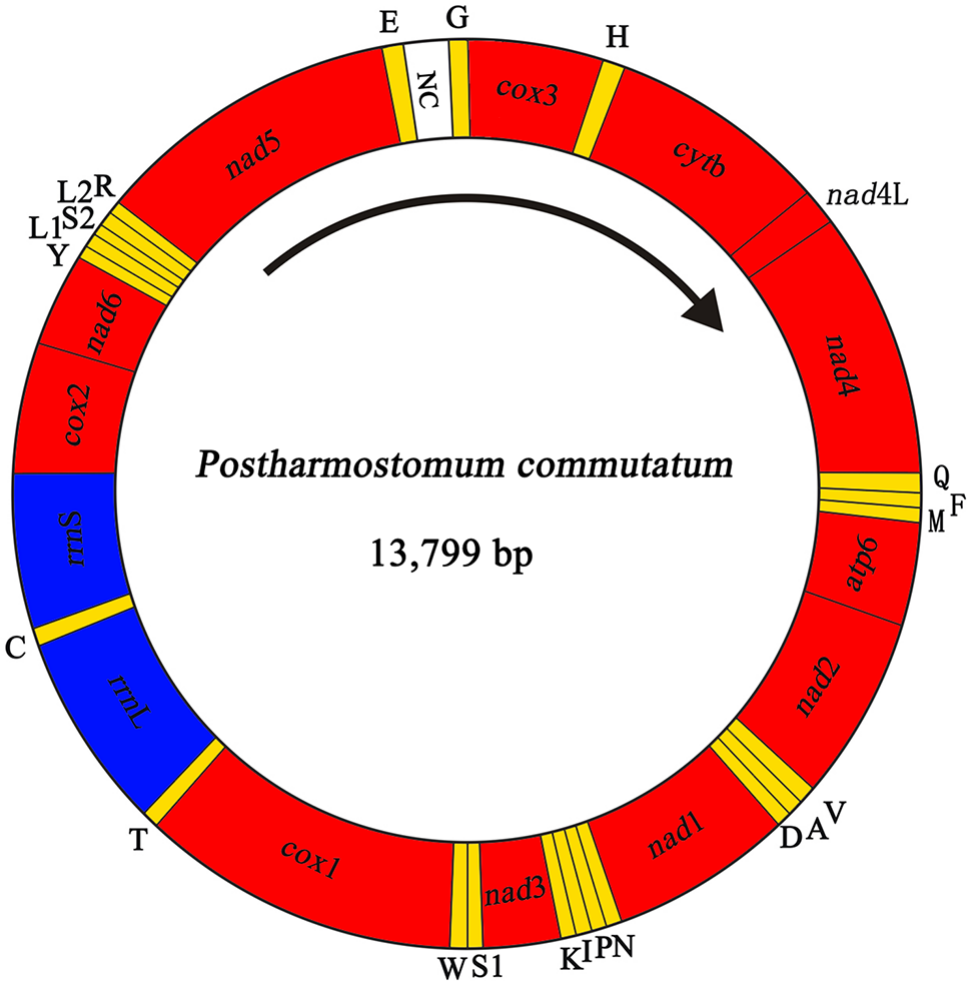
Organization of the mitochondrial genome of *Postharmostomum commutatum*. Scale is approximate. All genes have standard nomenclature except for the 22 tRNA genes, which are designated by the one-letter code for the corresponding amino acid, with numerals differentiating each of the two leucine- and serine-specifying tRNAs (L_1_ and L_2_ for codon families CUN and UUR, respectively; S_1_ and S_2_ for codon families UCN and AGN, respectively). All genes are transcribed in the clockwise direction. ‘NC’ indicates the non-coding region.

We sequenced the *P. commutatum* genome and produced over 3 Gb of Illumina short-read sequence datasets. A total of 14,070,228 × 2 raw reads with the size of 250 bp were generated and 13,411,012 × 2 clean reads were obtained for assembly of the mt genome. The entire mt genome sequence of *P. commutatum* (GenBank accession no. MN200359) was 13,799 bp in size (**Figure 1**). We further confirmed the completeness of mt genome assembly using five pairs of primers covering the whole 13,800 bp-long assembled sequence to amplify the entire mt genome of *P. commutatum*. All five fragments (∼2–4 kb each) were successfully confirmed by long PCR amplification (**Figure S2**). This complete mt genome was slightly shorter than some other digeneans (such as *Echinostoma caproni*, *Fischoederius elongatus* and *Schistosoma japonicum*) but was slightly longer than some digeneans (such as *C. complanatum*, *C. prussica* and *Opisthorchis viverrini*) (**Table 1**). This difference is mainly due to the total fraction of non-coding sequences. This circular mt genome contains 12 protein-coding genes (*cox*1-3, *nad*1-6, *nad*4L, *atp*6 and *cyt*b), 22 tRNA genes, two rRNA genes (*rrn*L and *rrn*S) and a non-coding (control or AT-rich) region (**Table 2** and **Figure 1**). The gene orders are the same as those of flukes of the order plagiorchiida, such as *O. viverrini*, *C. complanatum* and *Fasciola gigantica* (Cai et al., 2012; Liu et al., 2014; Chen et al., 2016), but distinct from those of blood flukes, such as *S. japonicum* (Zhao et al., 2009) and *S. turkestanicum* (Wang et al., 2011). Additionally, the mt genes of *P. commutatum* overlap by 47 bp in five locations (1 to 40 bp per location) (**Table 2**). The mt genome of *P. commutatum* has 15 intergenic regions, which range from 1 to 24 bp in size. The longest region is between *nad*4 and tRNA-Gln (**Table 2**).

**Table 2:** The organization of the mt genome of *Postharmostomum commutatum*.

Gene/Region	Positions	Size (bp)	Number of aa^a^	Ini/Ter codons	Anticodons	In
cox3	1–654	654	217	ATG/TAA		+8
tRNA-His (H)	663–729	67			GTG	+3
cytb	733–1,850	1,118	372	ATG/TA		0
nad4L	1,851–2,114	264	87	GTG/TAG		−40
nad4	2,075–3,370	1,296	431	ATG/TAA		+24
tRNA-Gln (Q)	3,395–3,455	61			TTG	−2
tRNA-Phe (F)	3,454–3,517	64			GAA	+1
tRNA-Met (M)	3,519–3,586	68			CAT	0
atp6	3,587–4,093	507	168	GTG/TAG		0
nad2	4,094–5,037	944	314	ATG/TA		0
tRNA-Val (V)	5,038–5,099	62			TAC	+2
tRNA-Ala (A)	5,102–5,164	63			TGC	0
tRNA-Asp (D)	5,165–5,229	65			GTC	0
nad1	5,230–6,144	915	304	ATG/TAG		+14
tRNA-Asn (N)	6,159–6,222	64			GTT	+1
tRNA-Pro (P)	6,224–6,290	67			TGG	0
tRNA-Ile (I)	6,291–6,355	65			GAT	−1
tRNA-Lys (K)	6,355–6,420	66			CTT	0
nad3	6,421–6,777	357	118	ATG/TAA		+3
tRNA-Ser^AGN^(S1)	6,781–6,839	59			GCT	+9
tRNA-Trp (W)	6,849–6,911	63			TCA	+12
cox1	6,924–8,498	1,575	524	ATG/TAG		+20
tRNA-Thr(T)	8,519–8,581	63			TGT	+1
rrnL	8,583–9,553	971				0
tRNA-Cys (C)	9,554–9,617	64			GCA	0
rrnS	9,618–10,349	732				+4
cox2	10,354–10,955	602	200	ATG/TA		0
nad6	10,956–11,403	448	149	ATG/T		0
tRNA-Tyr (Y)	11,404–11,468	65			GTA	0
tRNA-Leu^CUN^ (L1)	11,469–11,535	67			TAG	−3
tRNA-Ser^UCN^ (S2)	11,533–11,601	69			TGA	+17
tRNA-Leu^UUR^ (L2)	11,619–11,682	64			TAA	−1
tRNA-Arg (R)	11,682–11,747	66			TCG	+2
nad5	11,750–13,313	1,564	521	ATG/T		0
tRNA-Glu (E)	13,314–13,375	62			TTC	0
Non-coding region (NC)	13,376–13,733	358				0
tRNA-Gly (G)	13,734–13,799	66			TCC	0

a The inferred length of amino acid (aa) sequence of 12 protein-coding genes, Ini/Ter codons: initiation and termination codons; In, Intergenic nucleotides (between the current gene and the next gene).

The nucleotide composition of the complete mt genome of *P. commutatum* is biased toward A+T (64.8%), in accordance with mt genomes of other digeneans (Zhao et al., 2009; Wang et al., 2011; Suleman et al., 2019b). AT- and GC- skews are a measure of compositional asymmetry. In *P. commutatum* mt genome, AT- skews values were always negative, while the values of GC- skew were positive (**Table 3**). The AT- skew value observed is −0.306 on average, ranging from −0.448 (*nad*6) to −0.140 (*rrn*S). The average GC- skew value observed is 0.466, ranging from 0.339 (22 tRNA) to 0.737 (*nad*3) (**Table 3**). In all mt genome sequences of flatworm (including tapeworms, nematodes and trematodes) reported to date (Liu et al., 2013; Briscoe et al., 2016; Zhao et al., 2016), the GC skew is positive due to the very low C content in mt genomes.

**Table 3:** Nucleotide composition and skews of *Postharmostomum commutatum* mitochondrial genome.

Gene	Nucleotide frequency				A+T (%)	AT-skew	GC-skew
	A (%)	G (%)	T (%)	C (%)			
atp6	20.9	25.8	43.6	9.7	64.5	−0.352	0.454
cox1	20.4	25.3	44.2	10.1	64.6	−0.368	0.433
cox2	23.1	25.9	40.4	10.6	63.5	−0.272	0.419
cox3	20.5	24.3	45.3	9.9	65.8	−0.377	0.421
cytb	22.8	23.5	43.3	10.4	66.1	−0.310	0.386
nad1	19.7	29.8	43.4	7.1	63.1	−0.376	0.615
nad2	21.8	25.4	43.1	9.7	64.9	−0.328	0.446
nad3	18.2	29.7	47.6	4.5	65.8	−0.447	0.737
nad4	20.4	27.0	44.7	7.9	65.1	−0.373	0.547
nad4L	23.9	25.8	43.2	7.1	67.1	−0.288	0.564
nad5	19.4	28.2	44.8	7.6	64.2	−0.396	0.575
nad6	18.3	28.3	48.0	5.4	66.3	−0.448	0.680
rrnS	26.5	26.0	35.1	12.4	61.6	−0.140	0.354
rrnL 22 tRNA	27.4 27.0	24.7 24.7	37.2 36.1	10.7 12.2	64.6 63.1	−0.152 −0.144	0.395 0.339
Total	22.5	25.8	42.3	9.4	64.8	−0.306	0.466

### Protein-Coding Genes

A total of 3,405 amino acids are encoded by the *P. commutatum* mt genome. The enriched A+T content was reflected in the codon usage. In *P. commutatum* mt genome, Leu and Phe are the most frequently encoded amino acids, and Gln is the least frequent (**Table 4**). Individually, the most frequently used amino acid was TTT (Phe; 8.93%), followed by TTG (Leu; 7.76%), TTA (Leu; 6.91%) and GTT (Val; 5.15%) (**Table 4**). All of the 12 identified protein-coding genes begin with ATG (*cox*1, *cox*2, *cox*3, *cyt*b, *nad*1, *nad*2, *nad*3, *nad*4, *nad*5 and *nad*6) or GTG (*atp*6 and *nad*4L) as their start codons. Seven of the 12 genes appear to use TAA (*cox*3, *nad*4 and *nad*3) or TAG (*nad*4L, *atp*6, *nad*1 and *cox*1) as the stop codon, while the other genes end with incomplete codon TA (*cyt*b, *nad*2 and *cox*2) or T (*nad*5 and *nad*6). This is very common in worm mt genomes, such as tapeworms (Nakao et al., 2007), nematodes (Hu et al., 2002; Hu et al., 2003; Kim et al., 2006) and trematodes (Chang et al., 2016). It is hypothesized that the mRNAs ending in T or TA are converted to TAA by post-transcriptional polyadenylation (Ojala et al., 1981).

**Table 4:** Codon usage of *Postharmostomum commutatum* mitochondrial protein-coding genes.

Amino acid	Codon	Number	Frequency (%)	Amino acid	Codon	Number	Frequency (%)
Phe	TTT	305	8.93	Met	ATA	83	2.43
Phe	TTC	5	0.15	Met	ATG	102	2.99
Leu	TTA	236	6.91	Thr	ACT	43	1.26
Leu	TTG	265	7.76	Thr	ACC	5	0.15
Ser	TCT	89	2.61	Thr	ACA	25	0.73
Ser	TCC	8	0.23	Thr	ACG	17	0.5
Ser	TCA	24	0.7	Asn	AAT	51	1.5
Ser	TCG	31	0.91	Asn	AAC	2	0.06
Tyr	TAT	145	4.25	Lys	AAA	34	1.0
Tyr	TAC	37	1.08	Lys	AAG	65	1.9
Stop	TAA	3	0.88	Ser	AGT	95	2.78
Stop	TAG	4	1.12	Ser	AGC	8	0.23
Cys	TGT	96	2.81	Ser	AGA	63	1.85
Cys	TGC	3	0.09	Ser	AGG	44	1.29
Trp	TGA	56	1.64	Val	GTT	176	5.15
Trp	TGG	64	1.87	Val	GTC	12	0.35
Leu	CTT	45	1.32	Val	GTA	91	2.67
Leu	CTC	2	0.06	Val	GTG	135	3.96
Leu	CTA	13	0.38	Ala	GCT	74	2.17
Leu	CTG	7	0.21	Ala	GCC	4	0.12
Pro	CCT	48	1.41	Ala	GCA	30	0.88
Pro	CCC	3	0.09	Ala	GCG	33	0.97
Pro	CCA	17	0.5	Asp	GAT	63	1.84
Pro	CCG	14	0.41	Asp	GAC	5	0.15
His	CAT	46	1.35	Glu	GAA	26	0.76
His	CAC	7	0.21	Glu	GAG	51	1.49
Gln	CAA	14	0.41	Gly	GGT	138	4.04
Gln	CAG	18	0.53	Gly	GGC	13	0.38
Arg	CGT	39	1.14	Gly	GGA	63	1.85
Arg	CGC	2	0.06	Gly	GGG	75	2.2
Arg	CGA	17	0.5	IIe	ATT	107	3.13
Arg	CGG	12	0.35	IIe	ATC	7	0.21

Excluding abbreviated stop codons (TA and T). Stop = Stop codon.

### Transfer RNA Genes and Ribosomal RNA Genes

The sizes of 22 tRNA genes identified in mt genome of *P. commutatum*, ranged from 59 to 69 bp in length. A standard four-arm cloverleaf structure was inferred for most of the tRNA genes. However, the tRNA-Ser^AGN^ (S1) gene shows an unorthodox structure, with the paired dihydrouridine (DHU) arm missing, as usual in all parasitic trematodes (also seen in some cestodes and nematodes) (Nakao et al., 2002; Hu et al., 2003). Structures for tRNA-Cys (C) and tRNA-Ser^UCN^ (S2) often vary somewhat among the parasitic trematodes. A paired DHU-arm of these tRNA genes is not seen in *Haplorchis taichui*, *S. mansoni* (Blair et al., 1999; Lee et al., 2013), but it is present in *P. commutatum*. The *rrn*L gene of *P. commutatum* is located between tRNA-Thr and tRNA-Cys genes, and *rrn*S gene is located between tRNA-Cys and *cox*2genes. The sizes of the *rrn*L and *rrn*S genes for *P. commutatum* were 971 bp and 732 bp, respectively (**Table 2**). The A+T contents of the *rrn*L *rrn*S genes for *P. commutatum* are 64.6% and 61.6%, respectively. The sizes and A+T contents of the two rRNA genes for *P. commutatum* are almost similar to those of other digeneans sequenced to date, such as that of *Clonorchis sinensis*, *Paragonimus ohirai* and *Uvitellina* sp. (Cai et al., 2012; Le et al., 2019; Suleman et al., 2019a).

### Non-Coding Region

The mt genome sequences of flukes contain usually two non-coding regions (NC) of significant size difference (Yan et al., 2013; Le et al., 2019; Suleman et al., 2019b). However, In *P. commutatum* mt genome, there is only one NC (**Table 2** and **Figure 1**). The NC is located between the tRNA-Glu and tRNA-Gly, and lacks any tandem repeats. Its size is 358 bp and A+T contents is 76.5%. One NC was also identified in *H. taichu* and *Fasciolopsis buski* mt genomes (Lee et al., 2013; Ma et al., 2017). Although the function of non-coding regions is currently unknown, the high A+T content predicts an involvement in the initiation of replication (Keddie et al., 1998).

### Non-Synonymous/Synonymous Substitution Ratio of Protein-Coding Genes

**Figure 2:**
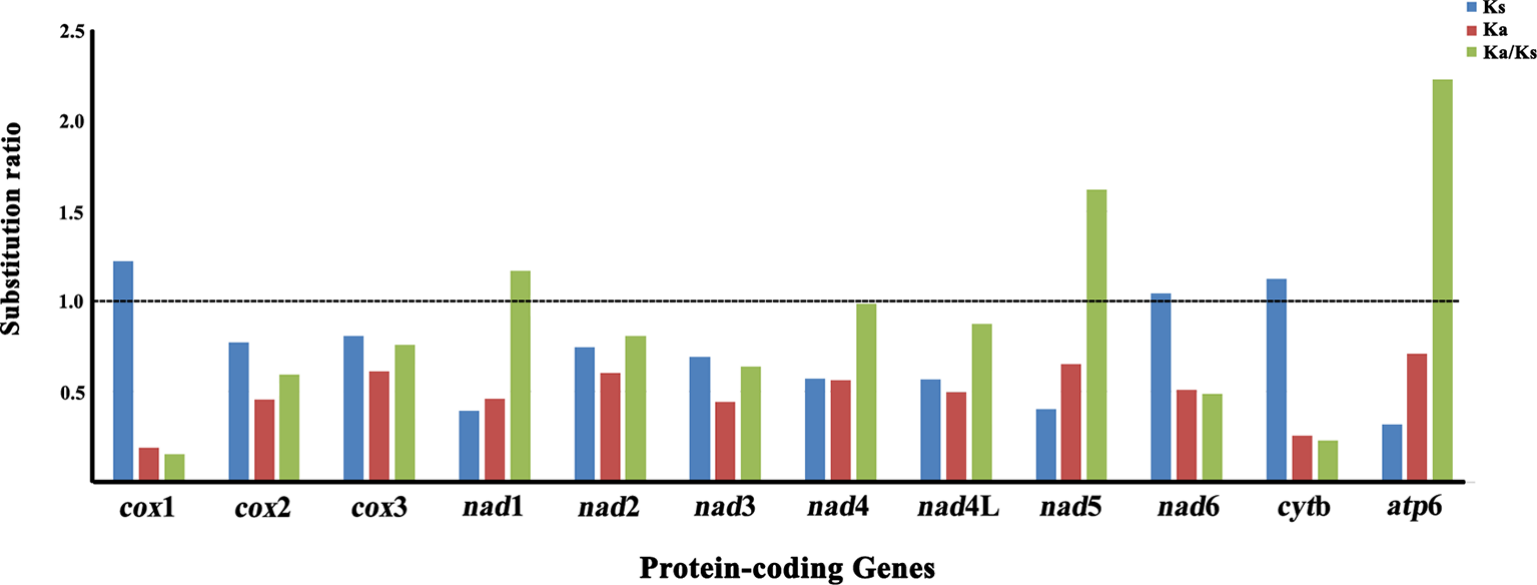
Substitution ratios in the mitochondrial genomes of digeneans. The rate of non-synonymous (Ka), the rate of synonymous (Ks) substitutions, and the respective ratios (Ka/Ks) for individual protein-coding genes are shown.

The non-synonymous (Ka)/synonymous (Ks) substitutions ratio is particularly useful for characterizing evolutionary relationships between mt protein-coding genes in closely-related species (Fay and Wu, 2003). The Ka/Ks ratio was calculated for nucleotide sequences of all 12 mt protein-coding genes of *P. commutatum* and other digeneans (**Table 1**). The Ka/Ks ratio is a measure of selective pressures acting on genes, which indicates either negative (Ka/Ks <1) or positive (Ka/Ks >1) or that positive and negative selection counter-balance each other (Ka/Ks = 1) (Meganathan et al., 2011; Li et al., 2012). In the *P. commutatum* mt genome, *atp*6 appeared to have the highest Ka/Ks ratio (2.230), while *cox*1 is the lowest Ka/Ks ratio (0.154) (**Figure 2**). Herein, the Ka/Ks ratio of nine protein-coding genes (*cox*1, *cox*2, *cox*3, *nad*2, *nad*3, *nad*4, *nad*4L, *nad*6 and *cyt*b) was <1 (range: 0.154 to 0.989), suggesting that these mt protein-coding genes of digeneans are under purifying selection. The Ka/Ks ratio of three protein-coding genes (*nad*1, *nad*5 and *atp*6) was >1 (range: 1.169 to 2.230), indicating that these mt protein-coding genes of digeneans have evolved under positive or Darwinian selection. A similar pattern is also observed for nematode mt genomes (Liu et al., 2013).

### Comparison With Other Selected Digeneans Mt Genomes

The amino acid sequences of *P. commutatum* were compared with other digeneans (**Table 5**). In addition, the amino acid sequence similarities between *P. commutatum* and three species from the order Plagiorchiida ranged from 22.6–72.1% (**Table 5**). However, the amino acid sequence similarity between *P. commutatum* and the selected three species from the order Diplostomida ranged from 20.2–71.0% (**Table 5**). These results show that the superfamily Brachylaimoidea (represented by *P. commutatum*) was more closely related to the members of order Plagiorchiida than it was to the members of order Diplostomida. Based on identity, COX1 was the most conserved protein, whereas ATP6 was the least conserved (**Table 5**).

**Table 5:** Pairwise identities (%) in mitochondrial amino acid sequences between *Postharmostomum commutatum* and other representative digeneans.

Gene/Genome	Diplostomida		Plagiorchiida			Diplostomida		
	CC	CP	EP	EM	OV	SH	SJ	TR
atp6	32.1	30.4	22.6	31.5	31.0	20.2	28.0	27.4
cox1	71.8	78.2	65.2	72.1	70.4	69.3	71.0	73.4
cox2	53.0	59.4	52.3	42.5	48.0	52.0	47.0	48.7
cox3	33.6	29.0	25.8	31.8	29.0	22.6	20.4	24.5
cytb	63.2	65.0	60.2	66.7	61.8	46.0	50.1	49.2
nad1	54.7	50.8	47.3	49.0	48.0	39.4	40.9	44.0
nad2	31.6	29.5	23.1	27.7	30.1	26.2	29.2	23.0
nad3	54.2	52.2	43.5	53.4	46.6	44.9	40.7	50.0
nad4	49.3	49.3	45.9	47.3	45.2	31.4	31.8	33.3
nad4L	58.6	59.8	46.0	62.1	55.2	30.2	33.3	31.4
nad5	46.3	47.0	35.7	52.0	36.7	32.4	30.9	31.9
nad6 EmtG	39.2 37.9	38.9 43.8	33.6 37.3	39.6 37.8	40.3 35.6	24.8 29.8	30.9 35.2	32.9 29.6

^†^Digeneans: CC, Clinostomum complanatum; CP, Cyathocotyle prussica; EP, Eurytrema pancreaticum; EM, Echinostoma miyagawai; OV, Opisthorchis viverrini; SH, Schistosoma haematobium; SJ, Schistosoma japonicum; TR, Trichobilharzia regenti; EmtG, entire mitochondrial genome.

### Phylogenetic Analyses

**Figure 3:**
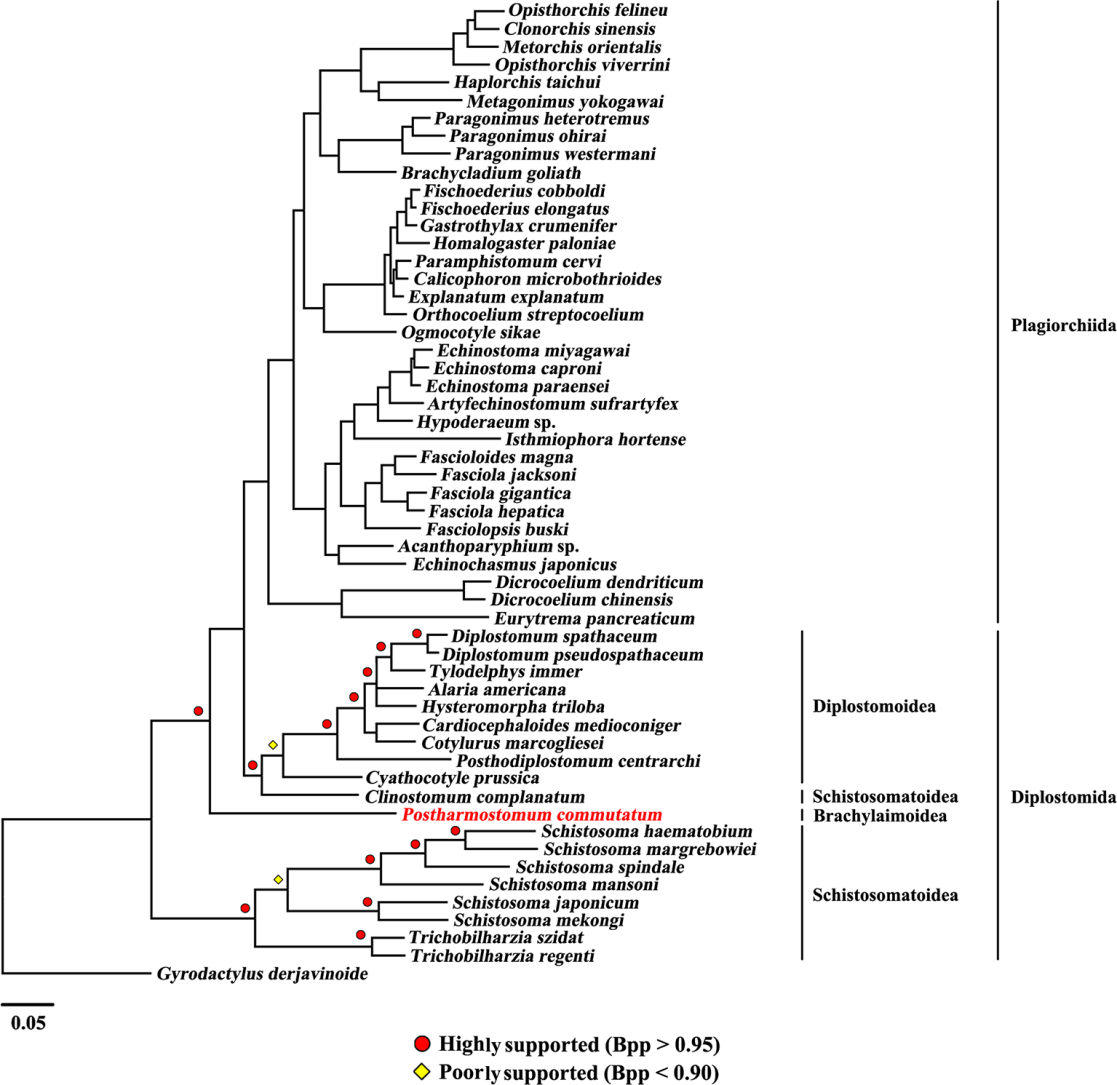
Phylogenetic relationships of *Postharmostomum commutatum* with other selected digeneans based on mitochondrial sequence data. The concatenated amino acid sequences of 12 protein-coding genes were subjected to analysis by Bayesian inference (BI) using *Gyrodactylus derjavinoides* as an outgroup. Bayesian posterior probabilities (Bpp) values are indicated.

**Figure 4:**
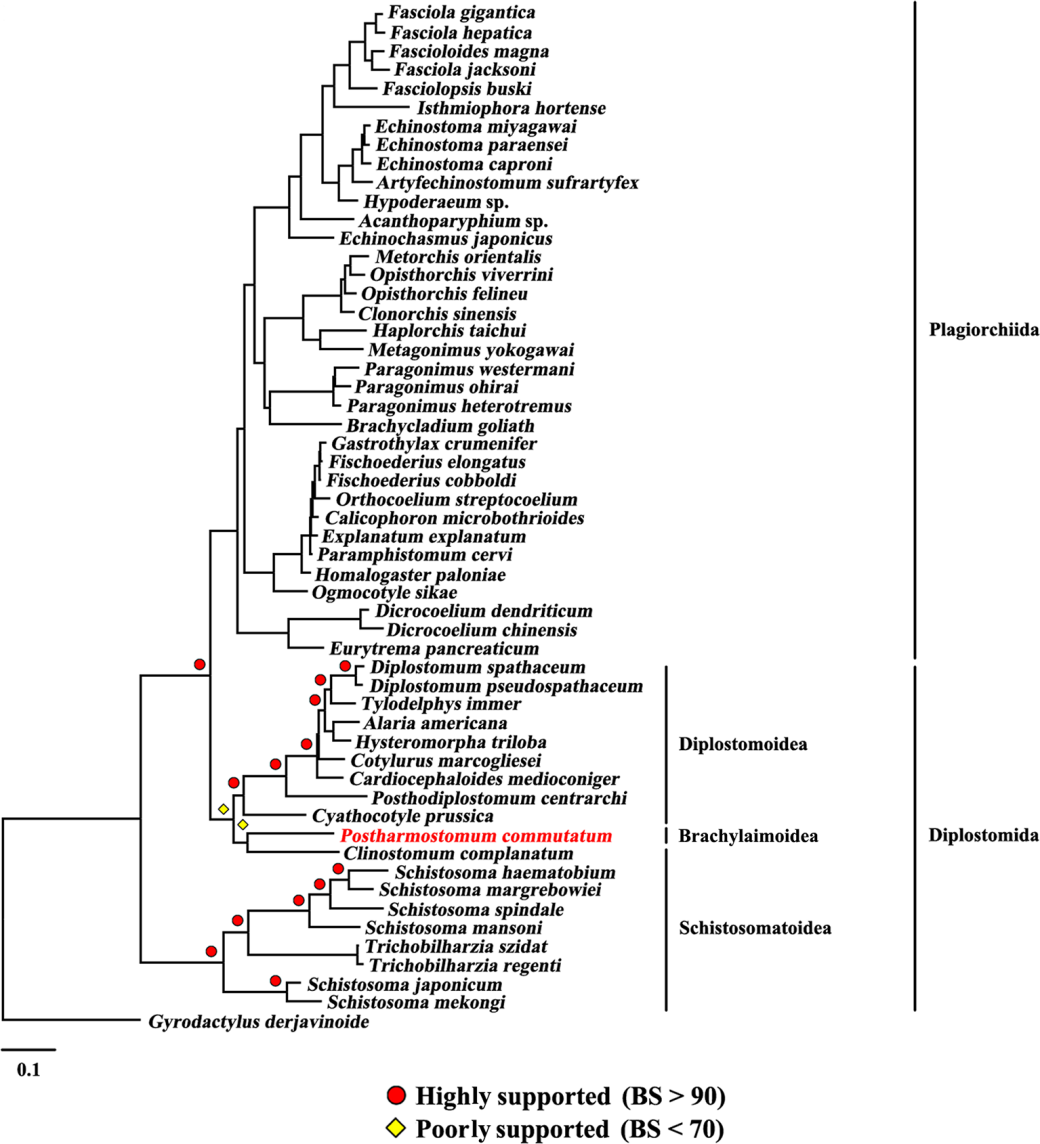
Phylogenetic relationships of *Postharmostomum commutatum* with other selected digeneans based on mitochondrial sequence data. The concatenated amino acid sequences of 12 protein-coding genes were subjected to analysis by Maximum likelihood (ML) using *Gyrodactylus derjavinoides* as an outgroup. Bootstrap frequency (BS) values are indicated.

**Figure 5:**
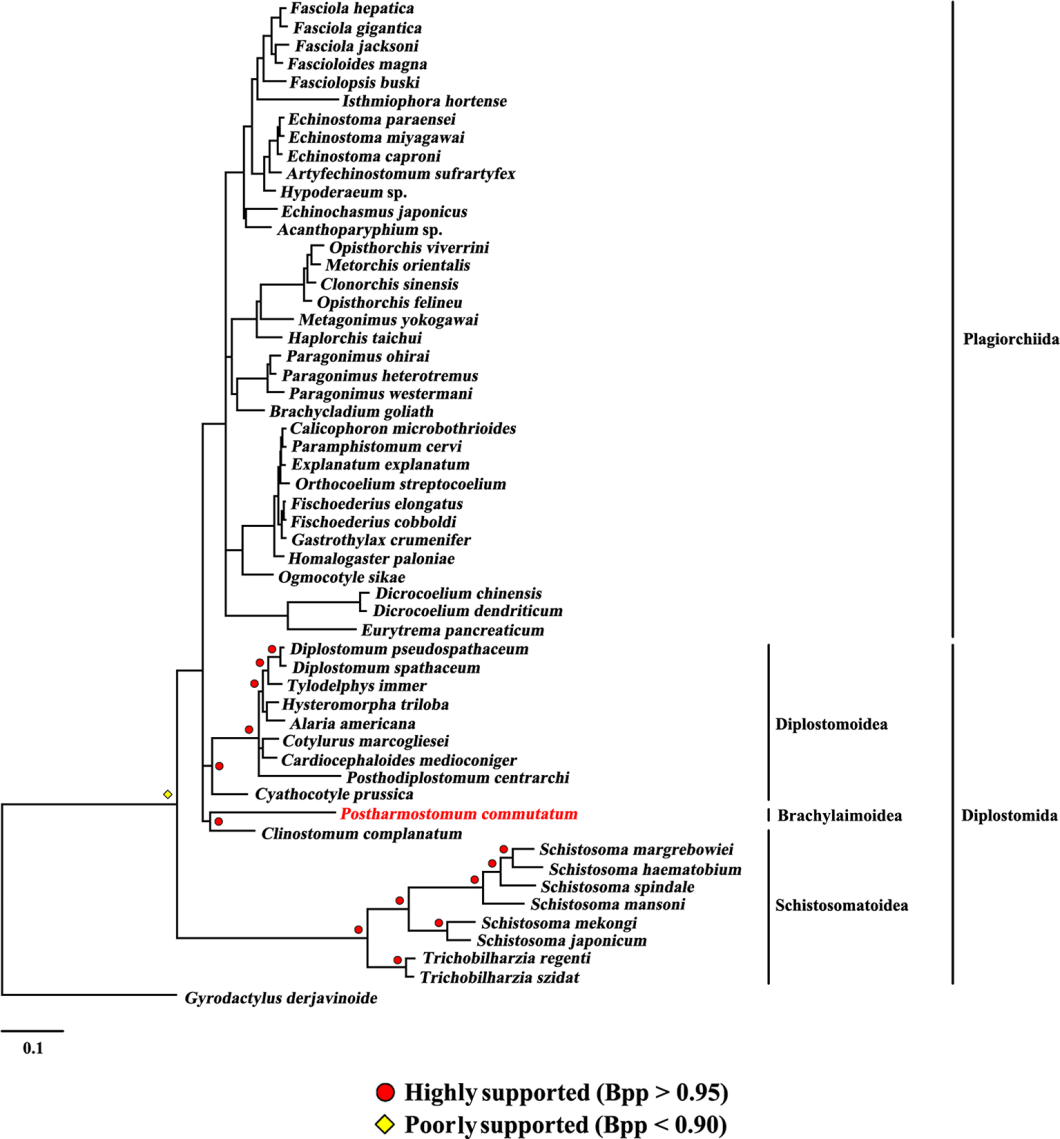
Phylogenetic relationships of *Postharmostomum commutatum* with other selected digeneans based on mitochondrial sequence data. The concatenated amino acid sequences of 12 protein-coding genes were subjected to analysis by PhyloBayes using *Gyrodactylus derjavinoides* as an outgroup. Bayesian posterior probabilities (Bpp) values are indicated.

The present study included three superfamilies (Diplostomoidea, Schistosomatoidea and Brachylaimoidea) from the order Diplostomida and phylogenetic analysis showed that the order Diplostomida was paraphyletic with strong support in BI (Bpp= 1.0, **Figure 3**) and ML (BS = 100, **Figure 4**), but was weakly supported in PhyloBayes (Bpp = 0.5, **Figure 5**) analyses. The monophyly of the superfamily Diplostomoidea was weakly supported with BI (Bpp = 0.87, **Figure 3**), and was strongly supported in ML (BS = 100, **Figure 4**) and PhyloBayes (Bpp = 1.0, **Figure 5**) analyses. The superfamily Schistosomatoidea, however, was not monophyletic in all of the three phylogenetic analyses in this study. One species (*C. complanatum*) from the superfamily Schistosomatoidea was more closely related to *C. prussica* (Diplostomoidea) (**Figure 3**) or *P. commutatum* (Brachylaimoidea) (**Figures 4** and **5**) than it was to the other 8 species from the superfamily Schistosomatoidea.

Eleven species representing three superfamilies (Diplostomoidea, Schistosomatoidea and Brachylaimoidea) from the order Diplostomida were more closely related to the members of the order Plagiorchiida than they were to the other eight species from the order Diplostomida. Our results were consistent with those of previous studies from mt genome datasets. For example, Brabec et al. (2015) sequenced the mt genomes of two species of diplostomids, and their phylogenetic analyses recovered the family Diplostomidae as the sister group of the order Plagiorchiida, although those relationships were supported by a low nodal support value. Chen et al. (2016) generated the complete mt genome of *C. complanatum* and performed a phylogenetic analysis with mt genomes, indicating that *C. complanatum* is the sister group of the order Plagiorchiida with strong support in ML analyses. Most recently, Locke et al. (2018) determined the complete mt genome of seven diplostomoids representing three families (Diplostomidae, Strigeidae and Cyathocotylidae) and their mt genome phylogenetic tree yielded the order Diplostomida as paraphyletic because strigeids, diplostomids and clinostomids were recovered as sister groups of the order Plagiorchiida, not the order Diplostomida. No species from this superfamily (Brachylaimoidea) of the order Diplostomida were included in previous analyses of mt genome datasets (Brabec et al., 2015; Briscoe et al., 2016; Chen et al., 2016; Locke et al., 2018). In the present study, the determination of the mt genome of *P. commutatum* allows a reassessment of the phylogenetic relationships of digeneans. Our results confirm and expand on recent analyses showing a paraphyletic pattern of mt genome evolution in the order Diplostomida (Brabec et al., 2015; Briscoe et al., 2016; Chen et al., 2016; Locke et al., 2018).

The work of Olson et al. (2003) created robustness and stability in higher systematics within the subclass Digenea based on nuclear rRNA genes. Monophyly of the order Diplostomida has also been established previously in nuclear rRNA genes (Olson et al., 2003; Littlewood et al., 2015; Locke et al., 2018; Pérez-Ponce de León and Hernández-Mena, 2019). The mt genomic phylogenetic relationships of the order Diplostomida revealed a conflict with the rDNA phylogeny. Locke et al. (2018) discussed the possible causes of the mt genome topology and noted that the discrepancy occurs along short internal branches at the base of longer terminal branches, which could be related to a rapid radiation and incomplete lineage sorting. Along these short internal branches, mt genomes of digeneans may have a lower phylogenetic signal than nuclear genomes, exacerbating effects of incomplete taxon sampling (Hedtke et al., 2006; Philippe et al., 2011). In addition, both mt and nuclear genome data for representatives of the Diplostomida and of the early divergent lineages of the Plagiorchiida are needed to address the relationships of the two major lineages of the Digenea (Locke et al., 2018). Although the number of digenean mt genome sequences is increasing, to date, mt genomes of many lineages of digeneans are underrepresented or not represented. Insufficient taxon sampling for digenean mt genomes might be the cause of the discordance between the mt and nuclear datasets. Therefore, more mt genomes of digenean species representing families that have not yet been sequenced should be included in future analysis to resolve the taxonomic problems of digeneans because mt genome sequences have been shown to resolve deep-level relationships in many metazoan groups (Littlewood, 2008) and the use of mtDNA sequences has been considered promising (Philippe et al., 2011).

## Conclusion

The present study determined the complete mt genome sequence of *P. commutatum*, which shares some similarity with, and interesting differences to, other digeneans. Phylogenetic analyses showed that *P. commutatum* was recovered as sister group of the order Plagiorchiida, supporting that the order Diplostomida is paraphyletic. The availability of the *P. commutatum* mt genome should represent a rich source of genetic markers for molecular epidemiological, population genetic and phylogenetic studies of parasitic flukes of socio-economic importance in poultry.

## Data Availability Statement

Publicly available datasets were analyzed in this study. This data can be found here: GenBank accession no. MN200359.

## Ethics statement

All procedures involving animals in the present study were approved and this study was approved by the Animal Ethics Committee of Hunan Agricultural University (No. 43321503).

## Author Contributions

G-HL and Y-TF conceived and designed the study, and critically revised the manuscript. Y-TF and Y-CJ performed the experiments. Y-TF and Y-CJ analyzed the data. G-HL and Y-TF drafted the manuscript. Y-TF and Y-CJ helped in study design, study implementation, and manuscript preparation. All authors read and approved the final manuscript.

## Funding

This study was supported by the Planned Program of Hunan Province Science and Technology Innovation (grant no. 2018RS3085) and the Training Program for Excellent Young Innovators of Changsha (grant No. KQ1802035).

## Conflict of Interest

The authors declare that the research was conducted in the absence of any commercial or financial relationships that could be construed as a potential conflict of interest.

## Supplementary Material

Table S1 Primers used for assembly validation.

Figure S1 Inferred phylogenetic relationships among *P. commutatum* and other relatives based on mitochondrial *cox*1 sequences utilizing maximum likelihood (ML) using *Ornithobilharzia canaliculata* as an outgroup.

Figure S2 PCR amplicons from the mitochondrial genome of *Postharmostomum commutatum*. Amplicons generated with the *P. commutatum* primers. M: DL5000 DNA marker, 1: Validation_01, 2: Validation_02, 3: Validation_03, 4: Validation_04, 5: Validation_05, 6: Negative control.
