# Supplementary material for: The Complete Mitochondrial Genome of the Caecal Fluke of Poultry, Postharmostomum commutatum, as the First Representative from the Superfamily Brachylaimoidea
Source: Front Genet. 2019 Oct 25;10:1037. doi: 10.3389/fgene.2019.01037 (PMC6823182; doi:10.3389/fgene.2019.01037)
Supplement: Table S1 — Primers used for assembly validation. [file Table_1.docx]

Supplementary Material

Table S1∣Primers used for assembly validation.

| Primer no. | Direction | Sequence (5'-3') | Size (bp) |
| --- | --- | --- | --- |
| Validation_01 | F | CAGGTGCTTGTAGCGAGTTAGAT | ~ 2300 |
|  | R | CAACAACGGAGTATTCCTCACCT |  |
| Validation_02 | F | GTTGGCTTGCTTGAGTAGGAGTA | ~ 2500 |
|  | R | CTACTTCGTCTCCTTCCCAACAT |  |
| Validation_03 | F | TATGTTGGGAAGGAGACGAAGT | ~ 2000 |
|  | R | ATAGAACCTCCCATACTGAAACC |  |
| Validation_04 | F | TAAAGAGGTGGGTAAGTCGCAGTG | ~ 3000 |
|  | R | ATTGATACAACGCTAAGTCCACC |  |
| Validation_05 | F | GTAGGGTGGACTTAGCGTTGTAT | ~ 4000 |
|  | R | ACCAACGCTTACCATCTAACTCG |  |
