# Supplementary figures and images for: The Complete Mitochondrial Genome of the Caecal Fluke of Poultry, Postharmostomum commutatum, as the First Representative from the Superfamily Brachylaimoidea
Source: Front Genet. 2019 Oct 25;10:1037. doi: 10.3389/fgene.2019.01037 (PMC6823182; doi:10.3389/fgene.2019.01037)

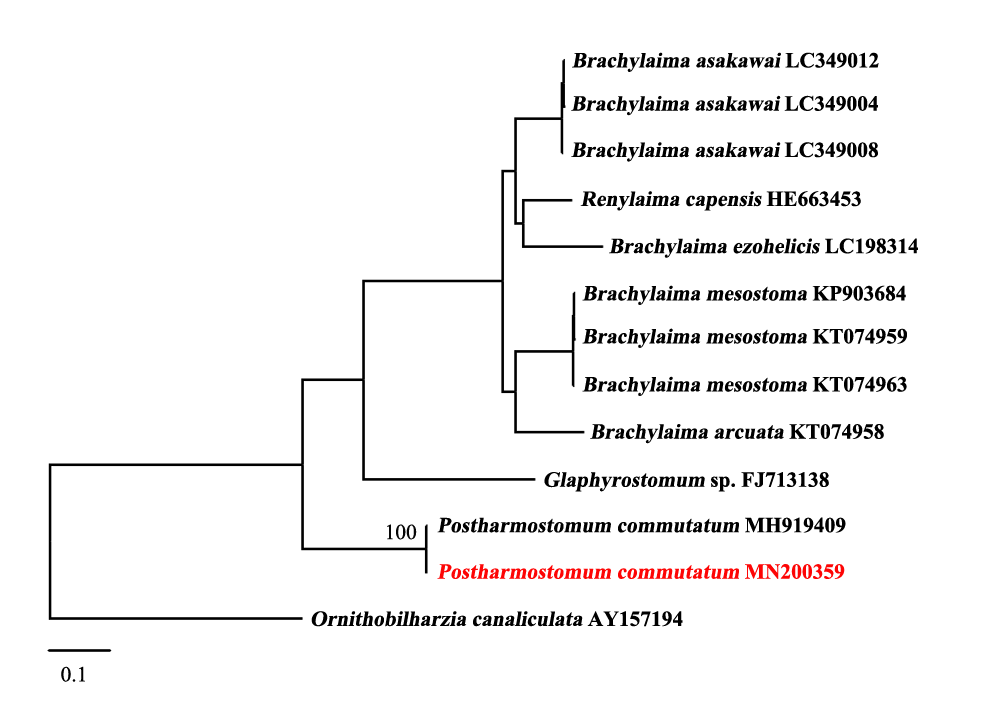

Supplement: Figure S1 — Inferred phylogenetic relationships among P. commutatum and other relatives based on mitochondrial cox1 sequences utilizing maximum likelihood (ML) using Ornithobilharzia canaliculata as an outgroup. [file Image_1.tif]
